# Planned missing data designs and methods: Options for strengthening inference, increasing research efficiency and improving animal welfare in ecological and evolutionary research

**DOI:** 10.1111/eva.13273

**Published:** 2021-07-22

**Authors:** Daniel W. A. Noble, Shinichi Nakagawa

**Affiliations:** ^1^ Division of Ecology and Evolution Research School of Biology The Australian National University Canberra ACT Australia; ^2^ Ecology and Evolution Research Centre School of Biological, Earth and Environmental Sciences The University of New South Wales Sydney NSW Australia

**Keywords:** data augmentation, hierarchical models, mixed effects models, multilevel modelling, multiple imputation, multiple working hypotheses, personality, quantitative genetics, reduction, refinement

## Abstract

Ecological and evolutionary research questions are increasingly requiring the integration of research fields along with larger data sets to address fundamental local‐ and global‐scale problems. Unfortunately, these agendas are often in conflict with limited funding and a need to balance animal welfare concerns. Planned missing data design (PMDD), where data are randomly and deliberately missed during data collection, combined with missing data procedures, can be useful tools when working under greater research constraints. Here, we review how PMDD can be incorporated into existing experimental designs by discussing alternative design approaches and demonstrate with simulated data sets how missing data procedures work with incomplete data. PMDDs can provide researchers with a unique toolkit that can be applied during the experimental design stage. Planning and thinking about missing data early can (1) reduce research costs by allowing for the collection of less expensive measurement variables; (2) provide opportunities to distinguish predictions from alternative hypotheses by allowing more measurement variables to be collected; and (3) minimize distress caused by experimentation by reducing the reliance on invasive procedures or allowing data to be collected on fewer subjects (or less often on a given subject). PMDDs and missing data methods can even provide statistical benefits under certain situations by improving statistical power relative to a complete case design. The impacts of unplanned missing data, which can cause biases in parameter estimates and their uncertainty, can also be ameliorated using missing data procedures. PMDDs are still in their infancy. We discuss some of the difficulties in their implementation and provide tentative solutions. While PMDDs may not always be the best option, missing data procedures are becoming more sophisticated and more easily implemented and it is likely that PMDDs will be effective tools for a wide range of experimental designs, data types and problems in ecology and evolution.

## 1 INTRODUCTION

Missing data are a widespread problem in ecological and evolutionary research (Ellington et al., 2015; Nakagawa, 2017; Nakagawa & Freckleton, 2010, 2011), often resulting in the exclusion of a substantial amount of available (but incomplete) data (e.g., through ‘complete case’ or ‘pairwise deletion’). This contributes to a reduction in statistical power and, if the nature of ‘missingness’ is not considered carefully, can lead to biased parameter estimates (Enders, 2001b; Graham, 2009; Little et al., 2013; Nakagawa & Freckleton, 2010). Theoretical frameworks for dealing with incomplete data have received substantial attention, and missing data theory is now a well‐developed field of research grounded on solid statistical theory (Enders, 2001a; Graham, 2003, 2009; Graham et al., 1996; Little et al., 2013; Little & Rubin, 2002; van Buuren, 2012). While social scientists commonly use missing data methods, these techniques remain relatively unknown, and seldom applied, in ecological and evolutionary circles (Nakagawa & Freckleton, 2010).

Making use of incomplete data is seldom discussed by ecologists and evolutionary biologists despite statistical tools being more widely available and easier to use than ever. In contrast, social scientists have made use of incomplete data for a long time—even embracing missing data during the design of experiments to help address fundamental research questions (Graham et al., 2006). Planned missing data design (PMDD) is an approach that involves deliberately planning to ‘miss’ data as part of an experiment and it has a long history (e.g., see Bose, 1939; and much of its development was done in the context of proficieny testing, see Johnson, 1992), in other words deliberately not collecting data on certain variables, time points or experimental subjects. While this seems like an odd thing to do, if missing data in the variables of interest is completely random, or can be made random, existing statistical frameworks are known to do an excellent job at estimating parameters and standard errors (Schafer & Graham, 2002; van Buuren, 2012). There are a number of potential benefits to using PMDDs in combination with missing data methods in the fields of ecology and evolution. However, ecologists and evolutionary biologists are likely unaware of their potential applications given it has developed in a disparate research field.

Here, we argue that missing data methods and PMDD have the potential to expand the scope, reduce research costs and alleviate animal welfare concerns, facilitating higher impact research. Our intention is to provide a greater appreciation for missing data at the design stages and overview some statistical tools that can be used to deal with random and nonrandom missing data. The latter is known to cause bias in model estimates and inferences (Little & Rubin, 2002; Nakagawa, 2017). Here, we briefly introduce missing data theory and then describe a few core statistical tools that can be used with incomplete data. We provide simulated case studies as supplemental examples, along with code, to show how missing data procedures can be used to yield valid inferences even with hierarchically structured data that is common in ecological and evolutionary research (Enders et al., 2016; Quartagno & Carpenter, 2016; Resche‐Rigon & White, 2018). We also describe alternative PMDDs, overviewing some of the different experimental designs that can be implemented, what they involve and important design considerations. Then, we overview some important benefits of using a PMDD and end with a discussion on some of the challenges and limitations to their use—providing suggestions for how these can be rectified.

## 2 A BRIEF INTRODUCTION TO MISSING DATA THEORY

Missing data patterns can generally be classified as falling into one of three different types—based on the different mechanisms generating missing data—missing completely at random (MCAR), missing at random (MAR) and missing not at random (MNAR) (Graham, 2009; Little & Rubin, 2002; Nakagawa, 2017; Nakagawa & Freckleton, 2010; Rubin, 1976; van Buuren, 2012). The distinction between these three missing data mechanisms is important to understand the implementation of missing data procedures and why PMDDs can be useful. Missing data (either in response or predictor variables) are considered to be MCAR when missingness is random with respect to both observed and unobserved (i.e., not collected in the study) data (Enders, 2001b; Nakagawa, 2017). In other words, the observed data are simply a random subsample of complete data (Enders, 2001b). In contrast, missing data are considered MAR when the missing values in a data set are correlated with values of other observed variables in the data set (Enders, 2001b; Graham, 2009). For example, if we were interested in understanding the correlation between survival to 1 year and mass at 6 months, we would find that individuals that die before 6 months are missing data on mass, but missing data on mass is correlated with individual lifespan, which is known. Missing not at random (MNAR) occurs when missing values are correlated with unobserved (uncollected) data or the missing values themselves. For example, we may be missing behavioural data on small‐sized animals within a population because they tend to be ‘shy’ and difficult to capture (e.g., Biro & Dingemanse, 2009), in which case we would be missing both behavioural and morphological data on a nonrandom sample of the population. Under these situations, dealing with incomplete data is difficult (possibly even impossible) because statistical techniques for estimating parameters when data are MNAR are difficult to implement given the need to explicitly model the process of missingness (Schafer & Graham, 2002).

Missing data mechanisms have different consequences for statistical results when incomplete data are excluded prior to analysis, as is often the case (i.e., referred to as ‘complete case’, ‘pairwise deletion’ or ‘listwise deletion’). While MCAR often results in a loss of power when data are excluded from an analysis, it does not bias parameter estimates (Enders, 2001b; Graham, 2009; Nakagawa & Freckleton, 2010; Schafer & Graham, 2002). In contrast, when missing data are MAR or MNAR, excluding data will result in both a loss of power and biased parameter estimates (sometimes severly so; Enders, 2001b; Graham, 2009; Nakagawa & Freckleton, 2010; Schafer & Graham, 2002). To better appreciate the impact missing data can have on sample size, assume that we have 10 variables, each containing 5% missing data, and a total complete data set of *n* = 1000. If we used all variables in a statistical model, we may need to exclude as many as 500 observations. Statistical techniques for dealing with missing data rely on the pattern of missingness being MCAR or MAR, and if this assumption is met, then bias and coverage in parameter estimates can be improved (Ellington et al., 2015; Enders, 2001b; Nakagawa, 2017; Nakagawa & Freckleton, 2010; Schafer & Graham, 2002; van Buuren, 2012).

## 3 STATISTICAL PROCEDURES FOR DEALING WITH MISSING DATA

Using incomplete data from a planned missing data design hinges on the ability of researchers to make use of statistical procedures for handling missing data (Enders, 2010; Graham et al., 2006; Little & Rubin, 2002). It is therefore pertinent that we briefly review existing missing data procedures and provide some guidance on their implementation. We do not discuss these topics in great depth as there are a number of accessible reviews and books on these subjects already (Allison, 2012; Enders, 2001b; Little & Rubin, 2002; McKnight et al., 2007; Nakagawa, 2017; Schafer, 1997; van Buuren, 2012), but we do provide a few simulated cases studies showing readers how to apply missing data procedures to multilevel data in the in Appendix S1 (see also https://doi.org/10.17605/OSF.IO/YZHRN). It is important to recognize that missing data methods have been around for some time. They range from quite simple approaches, such as mean substitution or simple regression‐based single imputation, to more modern missing data methods that we describe below (Graham, 2009). While simple methods exist, our review focuses on modern missing data methods because they result in unbiased parameter estimates and suitable coverage across a diversity of contexts (Enders, 2010; Graham, 2009; McKnight et al., 2007).

We view missing data methods as falling under two broad categories—those implementing model‐based (MB) techniques and those using multiple imputation (MI) with the help of Rubin's rules (Enders, 2010; Rubin, 1987). This categorization follows that of McKnight et al. (2007). While we acknowledge that these two categories have some overlap (e.g., MI still technically uses a model to impute missing data), we believe that they capture the major differences in the types of missing data procedures that can be applied.

**FIGURE 1:**
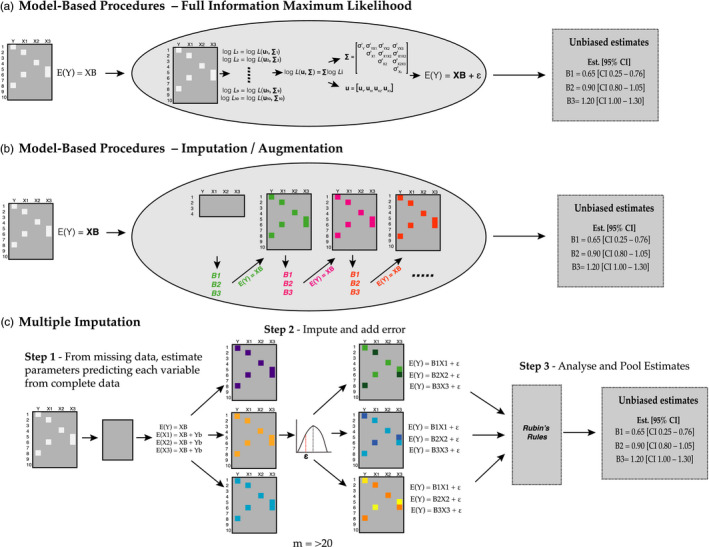
Two major types of missing data procedures: (a & b) model‐based procedures (e.g., full information maximum likelihood, expectation maximization, MCMC) and (c) multiple imputation procedures. Each large square represents a data set containing four variables (Y, X1, X2 and X3) and *n* = 10 observations. White squares represent missing data and grey squares complete data. Model‐based procedures can be broken into procedures. (a) The first procedure does not impute data but rather defines a case‐wise likelihood function [log *L*(u, Σ)] for each row of data that makes use of all available data that is present to estimate the maximum likelihood of a set of parameters (u, Σ) (i.e., full information maximum likelihood). (b) Alternatively, imputation and augmentation procedures take incomplete data in the analysis under a prespecified model [E(Y) = **XB**], augment missing data, estimate parameter estimates (B1, B2, B3) and then re‐iterate this process with updated parameters [different coloured B1, B2, B3 and E(Y) = XB] until the model converges on a set of unbiased parameter estimates. Multiple imputation (c) uses incomplete data and imputes missing measurements using regression equations with plausible values of missing data to generate *m* complete data sets. Note that all data can be used to impute missing measurements in each variable (hence, XB and Yb). To prevent downward bias in sampling variance for estimates of a given variable, residual error (based on the proposed error distribution of the variable being imputed) is added to each of the imputed data points (chequered small squares in step 2). These *m* data sets are then analysed with a given model, which can be different from the ones used to impute (hence why E(Y) can be used for each predictor in a separate model) and parameter estimates are pooled across data sets using equations proposed by (Rubin, 1987). Abbreviations are as follows: E = expectation of variable, or mean estimate of variable; **X** = design matrix; B = vector of parameter estimates (e.g., B1, B2, B3); **u** = a scalar vector describing the means for the multivariate distribution for each variable (Y, X1, X2, X3); **Σ** = the multivariate (co)variance matrix for Y, X1, X2, X3; log *L* = is the log likelihood; log *Li* = the log likelihood for row *i* of the incomplete data set; and *ε* = residual effect or random error

Model‐based procedures incorporate both observed and missing data into a single joint modelling approach. Some approaches do not actually impute missing data, but rather handle the missing data within a single model (e.g., full information maximum likelihood). Likelihood‐based methods use all available data to estimate parameters by defining a case‐wise likelihood function that works with each row of complete data, and the likelihood functions are then summed together to provide unbiased estimates of parameters and their uncertainty (Figure 1a—see Enders & Bandalos, 2001; Enders, 2010). Alternatively, some model‐based procedures augment/impute data proceeding through the following steps: (1) the parameters of a model are estimated using observed data; (2) parameters estimated in step 1 are then used to augment/impute missing data; and (3) model parameters are re‐assessed conditional on both observed and imputed data (Figure 1b; Nakagawa, 2017). These steps are re‐iterated until the model converges (Figure 1b). Model‐based approaches are advantageous in that they are fast, easily implemented (often under the assumption of multivariate normality; although alternative distributions could be assumed) and result in robust parameter estimates and standard errors (McKnight et al., 2007). They are also implemented during model fitting, meaning that there are no additional steps needed to deal with the missing data.

In contrast, multiple imputation proceeds by generating a set of *m* complete data sets where missing data are imputed using variables of interest. These *m* data sets can then be analysed normally (i.e., as if a complete data set existed) and the results (i.e., parameter estimates and standard errors) pooled (Figure 1c; Little & Rubin, 2002; Nakagawa, 2017; Schafer, 1997; Schafer & Olsen, 1998; van Buuren, 2012; van Buuren & Groothuis‐Oudshoorn, 2011), using Rubin's rules (Rubin, 1976). Usually, *m* = 40–50 imputed data sets perform well under a variety of situations (e.g., Nakagawa, 2017; Nakagawa & de Villemereuil, 2019), but this will depend on the models being estimated, and a larger number of imputed data sets will result in better quality estimates. Multiple imputation can better accommodate different error distributions (i.e., Bernoulli and Poisson) and allows practitioners to analyse the data in any way they wish using any software. It also separates the imputation and analysis steps allowing for different imputation and analysis models (Enders, 2010; McKnight et al., 2007) (although some multivariate Bayesian model‐based approaches exist to get around this limitation). However, it does require the additional steps of having to generate complete data sets prior to analysis followed by the need to subsequently pool the parameter estimates and standard errors. Nonetheless, with large sample sizes, and assuming statistical assumptions are met, MB and MI procedures are equally good at producing unbiased parameter estimates and their uncertainty (Allison, 2002).

### 3.1 Auxiliary variables to aid missing data methods

Auxiliary variables are variables that are not necessarily of interest with respect to the biological question at hand, but that are correlated with other variables and thus are predictive of missing measurements in these variables (Collins et al., 2001; Graham, 2003). Including auxiliary variables, especially ones that are expected to be predictive of missing data, has been shown to improve the accuracy and stability of estimates and to reduce their standard error (Allison, 2012; Enders, 2010; von Hippel & Lynch, 2013). The best auxiliary variables are those that are easy and cheap to collect and that are strongly correlated with a number of other variables within the data set (Collins et al., 2001; Graham, 2003; von Hippel & Lynch, 2013). Collins et al. (2001) have shown that auxiliary variables can be particularly useful when they change the missing data mechanism from MNAR to MAR and when the correlation between auxiliary variables and the response is high (*r* > 0.7). Adding even just 2–3 auxiliary variables can improve missing data procedures, and for the most part, an inclusive analysis strategy where a large number of auxiliary variables are included in the analysis is recommended (Enders, 2010, p. 128). However, the inclusion of too many (>10) can start to lead to a downward bias in regression coefficients and a decrease in precision (Hardt et al., 2012). While the number of auxiliary variables will depend on the specific study questions being asked, it is useful to include auxiliary variables with moderate‐to‐high correlations (>0.4) because they will be most effective in dealing with missing data. These will be particularly important where unplanned missing data exists (see Section 6.1 below) to increase the chances that assumptions of missing data methods are satisfied (i.e., MAR).

Experiments in ecology and evolution often collect variables that are not necessarily of interest, but can be used as auxiliary variables. Auxiliary variables, such as body dimensions, sex, age or even spatial data, can be included in missing data procedures (e.g., MI) to help deal with unplanned missing measurements. These will likely improve the performance of missing data procedures more generally. Variables do not necessarily need to be incorporated in models when testing the biological questions and hypotheses of interest (if using multiple imputation) (Graham, 2003). As an illustrative example, consider a field study on birds, where the spatial position (i.e., latitude and longitude) of nest boxes is known and stable through time. Here, the spatial position may not be of interest to researchers, but it may be the case that it is correlated with behaviour (e.g., shyness) and/or body mass because subordinate animals get pushed to the fringes of habitat by dominant individuals. As a consequence, they tend to be harder to recapture on repeated samples increasing the amount of missing data for these animals (Holtmann, Santos, Lara & Nakagawa, 2017). One way to use spatial coordinates might be to generate a spatial covariance matrix between observations and extract from the spatial covariance matrix its principal components (PCs). Multiple imputation could then make use of the PCs to impute missing data (e.g., using *mice* or *mi*—Table 1) for individuals that were not measured on a given sampling occasion. Similar approaches have been developed that make use of phylogenetic covariance matrices (Nakagawa & de Villemereuil, 2015) as well as the relatedness matrices (Hadfield, 2008).

**TABLE 1:** Examples of common packages and statistical programs that can be used to deal with missing data

Package	Prog.	Algorithms	Response/Predictor	Multilevel	Reference/link
*mi*	R	MI	B	Y	Su et al. (2011)
*mice*	R	MI	B	Y	van Buuren and Groothuis‐Oudshoorn (2011)
*micemd*	R	MI	B	Y	Audigier and Resche‐Rigon (2017)
*miceadds*	R	MI	B	Y	Robitzsch and Grund (2020)
*lavaan*	R	MB	B	Y	Rosseel (2012)
*blavaan*	R	MB	B	Y	Merkle and Rosseel (2018)
*jomo*	R	MI	B	Y	Quartagno and Carpenter (2016)
*Amelia*	R	MI	B	Y	Honaker et al. (2011)
*multimp*	R	MI	B	Y	https://github.com/inbo/multimput
*JAGS*	SA/R	MB	B	Y	https://mcmc‐jags.sourceforge.io
*MCMCglmm*	R	MB	R	Y	Hadfield (2010)
*brms*	R	MB/MI	B	Y	Bürkner (2017, 2018)
*ASReml*	SA/R	MB	B	Y	Butler (2009)
*SAS*	SA	MB/MI	B	Y	https://stats.idre.ucla.edu/sas/seminars/multiple‐imputation‐in‐sas/mi_new_1/
*SPSS*	SA	MB/MI	B	?	https://www.ibm.com/ms‐en/marketplace/spss‐missing‐values
*MPlus*	SA	MB/MI	B	Y	https://www.statmodel.com/index.shtml

Abbreviations: ?, unknown; B, both; MB, model‐based approaches; MI, multiple imputation; R, response; SA, stand‐alone program; Y, yes.

## 4 PLANNED MISSING DATA DESIGNS AND THEIR APPLICATION IN ECOLOGY AND EVOLUTION

Planned missing data designs involve deliberately collecting incomplete data sets by randomly missing measurements on subjects or missing measurement occasions from repeated observations of subjects (Graham et al., 2006; Little & Rhemtulla, 2013; Rhemtulla & Little, 2012). Researchers can then use model‐based or multiple imputation techniques to deal with incomplete data to answer their question(s). As a recent example, Herrera (2019) applied a simple PMDD to understand changes in pollinator abundance over 21 years across 65 plant species. While the study did not use missing data procedures, Herrera (2019) did plan to deliberately, but randomly, miss specific species–year combinations when sampling pollinator abundance. Importantly, any PMDD should always conform to the MCAR assumption because missing data are random by virtue of the experimental design. This feature makes it ideal for use with the various statistical methods designed to deal with incomplete data (Little & Rhemtulla, 2013). While a simple PMDD could randomly miss measurements across variables throughout the entire data set, there are a number of alternative designs that may also be useful that we discuss below.

### 4.1 Subset measurement design

Planned missing data design was first developed to deal with participant fatigue when answering questions during research surveys. It is particularly useful when there are also logistical and financial constraints to asking many different questions (Graham et al., 1994, 2006). For example, a common type of PMDD called the *multi‐form design* (MFD) involves creating alternative questionnaires that each contain overlapping questions and a sample of new questions (Graham et al., 2006; Little & Rhemtulla, 2013). Combining data on participants across the questionnaires, and then treating the questions participants were not given as missing information, allows parameters to be estimated based on the covariance between known answers (Graham et al., 2006).

**FIGURE 2:**
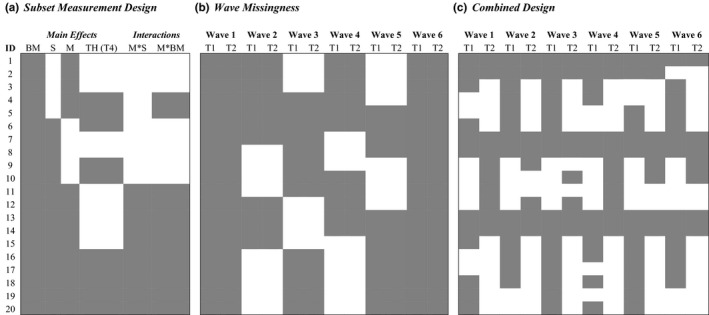
Three examples of planned missing data designs relevant for ecological and evolutionary research. In all cases, 20 individuals are shown along the rows (ID) and variables (e.g., BM) or traits (e.g., T1) measured are shown along columns. Abbreviations: BM = body mass; S = sex; M = metabolism; TH = thyroxine (T4); T1 = Trait 1; T2 = Trait 2. ‘Grey’ filled boxes indicate that traits on individuals are measured, and ‘white’ filled boxes are missed data. (a) Subset measurement designs randomize a set of variables to be measured on a sample of individuals. Body mass (BM) is strongly correlated with all three other variables and so is measured on all individuals in the study. Molecular determination of sex is needed with our species, and along with thyroxine, can both be costly to quantify so these traits are measured on a sample of individuals. Metabolism can also be time‐consuming to measure, and so, it is only quantified on a subsample of animals. While we can estimate all main effects (i.e., single parameter of interest) with this design, if interactions are of interest then one should have a PMDD that ensures there is enough data to effectively estimate the interaction parameters. (b) Wave missing design can be applied to longitudinal data. Here, only two traits are quantified once a month on 20 individuals. In each measurement wave (i.e., a month), a random sample of individuals is completely missing data on both traits. (c) Combined designs can miss certain individuals and also impose trait‐level missingness

Questionnaires are seldom used in ecology and evolutionary biology to collect data (aside from the field of ethnobiology; see Albuquerque et al., 2014). An analogous design is what we refer to as a *subset measurement design* (SMD) (Figure 2a). Similar to the MFD, a SMD involves quantifying a common set of variables across all individuals (e.g., body size) and then randomly allocating subjects to be measured on a subset of other variables (e.g., hormone concentrations and metabolism) (Figure 2a). Common variables can be those that are easily or cheaply measured, such as body size indices (e.g., mass, body/wing length). In contrast, variables that are expensive or logistically challenging to quantify (e.g., gene expression, hormone concentrations) can be randomly sampled on a subset of subjects during the experiment. When using incomplete data under a SMD, one should also consider, *a priori*, any potential interactions (Figure 2a) of interest and whether the planned missingness provides sufficient power to test these interactions (Enders, 2010).

### 4.2 Two‐method design

Subset measurement design can also be applied to situations where researchers have a choice between two variables that might both be indirectly related to some response variable. For example, one variable might be more easily and cheaply quantified but has larger measurement error. In contrast, the second variable is more logistically challenging to measure but is considered the ‘gold standard’ (i.e., lower measurement error/more informative to the question). The latter design is referred to as a *two‐method design* (TMD) in the social sciences (Little & Rhemtulla, 2013) and is used when one variable is known to be systematically biased (Graham et al., 2006). These approaches can be used to help understand the relationships between variables that are costly to collect (but often accurate) by allowing one to instead replace these measurements with cheaper (but often noisier) variables that are correlated with the expensive measurement variables. For example, we may be able to measure metabolic rate (rather cheap, but noisy) instead of concentrations of thyroxine (T4) (expensive) to understand the relationship between metabolic hormones and reactive oxygen species (ROS) (e.g., Figure 2a—where M is measured more than T4). Thyroxine concentrations are expected to have a more direct effect on ROS compared to metabolic rate because it is expected to modulate cellular metabolism directly. In contrast, metabolic rate measures aerobic respiration (i.e., often indirectly by measuring gas exchange), which can be impacted by LEAK respiration rates at the cellular level (Koch et al., 2021).

### 4.3 Longitudinal/wave missingness designs

Longitudinal research questions, where repeated measurements on a set of independent individuals are of interest, can use a PMDD called *wave missingness* (Figure 2b). Here, a random group of experimental subjects are not measured at all at a given time point or measurement occasion (Little & Rhemtulla, 2013; Rhemtulla et al., 2014). Waves can also be blocked such that all subjects are measured at the beginning and end measurement wave (e.g., months 1 and 6—Figure 2b) with subsamples of animals not measured at all in middle waves (i.e., pseudo‐randomized missingness; Rhemtulla et al., 2014; Rhemtulla & Little, 2012; e.g., month 2–5 in Figure 2b). Wave missingness designs have the potential to drastically decrease data collection costs. For example, we may be interested in understanding seasonal changes in two hormones by sampling the same set of individuals at monthly intervals. Sampling animals across all occasions means that we would need to sample blood 120 times and run 240 different assays. In contrast, using the missingness pattern in Figure 2b, we can reduce the total number of blood samples by ~20% and run 28% fewer assays (*n* = 66 fewer assays). In addition, a wave‐missingness design might be applied to ecological research questions to offset exspensive and time consuming field surveys. Interestingly, a TMD and SMD can also be applied in combination with longitudinal designs, and simulations have shown this to be an effective PMDD (Garnier‐Villarreal et al., 2014). The specific wave missingness design used will largely depend on the research question and the constraints faced in executing the study. Careful attention needs to be paid when using wave missingness designs as they may not perform well when sample sizes are small (Rhemtulla & Hancock, 2016). However, they can still be a valuable PMDD when resources are limited and there are ethical concerns about repeated measurements on the same subjects.

### 4.4 Considerations when using missing data designs

We have overviewed three of the more common designs that can be applied to experimental systems. However, PMDDs can be diverse, and combinations of the designs described above are probably necessary in many real research situations (e.g., Figure 2c) (Enders, 2010; Little & Rhemtulla, 2013; Rhemtulla et al., 2014; Rhemtulla & Little, 2012). Regardless of which PMDD is used, missing data should be avoided as much as possible in variables that are most pertinent to the specific hypothesis being tested or those that are expected to have small effect sizes (i.e., having complete measurements on these variables; Graham et al., 2006). It should be kept in mind that data sets with variables that are more strongly correlated with each other (i.e., *r* > 0.5) will also permit higher levels of missingness.

While PMDDs can be powerful tools, it is still unclear what designs work best at estimating parameters and standard errors across various situations. Despite simulations showing the benefits of PMDD, missing data itself can affect the information content in the data that is available for estimating parameters and standard errors (Rhemtulla & Hancock, 2016). It is important to recognize that missing information is not a simple function of the amount of missing data, it also depends on which variables are missing and the pattern of missingness (Rhemtulla & Hancock, 2016). There are many excellent resources evaluating the power and efficiency of different PMDDs under different contexts, and we refer interested readers to these for more detail (Enders, 2010, p. 23–36; Enders et al., 2016; Graham et al., 2006; Grund et al., 2016; Grund et al., 2017; Grund et al., 2018; Lüdtke et al., 2017; Resche‐Rigon & White, 2018; Rhemtulla & Hancock, 2016; Rhemtulla et al., 2014).

## 5 BENEFITS OF A PLANNED MISSING DATA DESIGN WITH MISSING DATA PROCEDURES

Planned missing data designs, combined with missing data procedures, can allow researchers to take advantage of the covariance among variables within a data set to expand the quality and scope of a study, reduce research costs and improve animal welfare outcomes. Strategic implementation of a PMDD can allow researchers to get a ‘bigger bang for their buck’ in terms of the research cost to outcome ratio, particularly for experiments involving expensive biochemical, proteomic, metabolomic and genomic work. Additionally, combining measurements of different variables in a single study will allow for greater power in distinguishing predictions from alternative hypotheses offsetting the ‘fallacy of the factorial design’ problem (Betini et al., 2017).

Planned missing data design can also provide significant advantages for animal welfare, allowing researchers to minimize pain, suffering and distress caused by experimentation (Cuthill, 2007). This could be achieved by using less invasive procedures, collecting data on fewer experimental subjects or less often on a given subject. The reduced burden on animals may even result in higher quality data because there is less potential to invoke stressful procedures repeatedly which may compromise animal function and impact how animals respond when re‐measured at later dates. For example, carp subjected to daily handling stress are more susceptible to blood flagellate infection resulting in reduced survival (Saeij et al., 2003). Such effects could limit the types of questions that can be answered by a given study. Interestingly, application of MFDs for behavioural intervention studies in psychology has been shown to result in higher quality data by reducing respondent fatigue in repeated questionaries (Harel et al., 2015). We expect these benefits to apply when using two‐method/subset measurement designs in ecology and evolutionary biology.

As mentioned above, missing data procedures can also lead to improved statistical power relative to using a complete case analysis and improve model convergence (Little & Rhemtulla, 2013). Planning on missing data before embarking on an experiment can also help counteract problems associated with nonrandom missing data, which can cause major bias and inferential problems (Little & Rubin, 2002; Nakagawa, 2017). When applying missing data procedures, adding in new variables that may be correlated with missingness will result in data that is MAR. This will help to correct for nonignorable missingness that are issues for complete case analyses.

As one example of how missing data approaches can improve coverage (i.e., estimation of standard errors), we conducted a simple simulation to compare model‐based missing data methods to a complete case design under varying sample sizes and levels of missing data. Many research fields, from quantitative genetics to behavioural ecology, are regularly interested in using multivariate mixed models to estimate between‐individual correlations between two phenotypic traits measured repeatedly (Careau & Wilson, 2017; Dingemanse & Dochtermann, 2013). Our simulation applies a simple PMDD in these contexts to understand how the between‐individual correlation estimate, along with its uncertainty, is affected (Section 5 in Appendix S1 – also found at https://doi.org/10.17605/OSF.IO/YZHRN). We show that, in this case, using MB approaches can improve upon the estimation of standard errors of between‐individual correlation coefficients compared to a complete case scenario (Figure S5.1 in Appendix S1). Detailed simulation studies on a range of different questions and problems have shown the circumstances under which different PMDDs can provide statistical benefits (Graham et al., 2006; Little & Rhemtulla, 2013) and where they can fail (Lüdtke et al., 2017; Rhemtulla & Hancock, 2016; Rhemtulla et al., 2014). While there is no replacement to obtaining complete data, these studies will be useful for weighing up the costs and benefits of a PMDD approach for a given study.

In summary, the application of PMDD with missing data methods deviates from normal experimental design considerations (e.g., should one collect data on 10 animals 10 times or 20 animals five times) in that it allows researchers to take advantage of the covariance between variables within their incomplete data to (1) incorporate more variables into a single analysis to test predictions from competing models without compromising the estimation of parameters and their uncertainty; (2) provide an ability to select less expensive (or less in invasive) variables and substitute them with correlated variables that are easier to collect but capture the relationships of interest; and (3) help overcome nonrandom missing data, which can impact results from complete case analyses.

## 6 CHALLENGES IN IMPLEMENTING PLANNED MISSING DATA DESIGNS AND METHODS

As with any new research method, there will be challenges, particularly in establishing the most suitable approaches that work across a wide diversity of different research questions and experimental designs. In addition, PMDD can be more work to implement as careful thought needs to be given to how missing data will impact the inferences drawn. The statistical procedures to deal with missing data will also mean that more work and attention needs to be paid to analysing incomplete data. Finally, application of missing data procedures to PMDDs is not always going to improve statistical inferences relative to complete case designs. Some simulations show they can be less efficient and even perform more poorly (Rhemtulla & Hancock, 2016). Nonetheless, given PMDD is still new, stimulating interest in them will be the first step to identifying problems and implementing solutions to any challenges. In addition, a PMDD could still be used without applying missing data procedures—assuming the nature of missingness is random, normal statistical procedures will still apply (e.g., see Herrera, 2019). Below we discuss some of the hurdles we see to implementing a PMDD and suggest some tentative solutions.

### 6.1 Unplanned missing data

As with any experiment, unplanned missing data will creep into PMDDs, such as when a piece of equipment malfunctions or when recording errors are identified leading to missing measurements that were unexpected. Random instances of missing data, even if unplanned, will not often affect statistical approaches to deal with incomplete data or the utility of PMDD unless missing data levels begin to get quite high. However, simulations show (e.g., Enders, 2010; Graham et al., 2006) that missing data procedures can perform quite well even with large amounts of missing data (see also Supplement S5 in Appendix S1). Nonetheless, there are real situations where unplanned missing data can be MNAR and this will affect any experiment regardless of whether a PMDD is implemented or not. We have outlined above how data can be made MAR by collecting auxiliary variables. Unplanned missing data can then be dealt with normally, along with any planned missing data, using the same statistical methods. Collecting auxiliary variables that are cheap and easy to collect when possible may help counter unplanned missing data.

### 6.2 Missing data procedures with complex models

Multiple imputation and model‐based procedures both work well with normally distributed data; however, in reality, variables often are non‐normally distributed and data are hierarchical in nature. While most statistical packages making use of MB procedures assume multivariate normality, MI procedures can also work with non‐normal data fit using generalized linear mixed effect models (e.g., Poisson GLMMs) (Schafer, 1997). We show an example case study of how MI can work with multilevel count data in the supplemental examples (Section S4 in Appendix S1—also found at https://doi.org/10.17605/OSF.IO/YZHRN). However, implementation in the context of GLMMs is still under active development and, in many cases, is restricted to simple random effect structures (Audigier & Resche‐Rigon, 2017; Enders et al., 2016; Quartagno & Carpenter, 2016; van Buuren & Groothuis‐Oudshoorn, 2011). Additionally, it is important to capture the hierarchical structure (in the case of mixed models) in the data along with any hypothesized interactions when applying missing data procedures. This may be challenging at times (Lüdtke et al., 2017). Nonetheless, two‐level random regression models can be run in a number of existing packages (e.g., *mice*, *miceadds*); however, they are currently limited to one random factor. Despite this, we believe that the capacity to run more sophisticated models will grow in the near future.

### 6.3 Overcoming psychological barriers to missing data

One of the biggest challenges to implementing PMDDs probably involves the need for researchers to overcome the ‘psychological taboos’ around missing data and the suspicion of techniques for handling incomplete data (Enders, 2010). Unplanned missing data are already treated with a sense of disdain and annoyance by ecologists and evolutionary biologists. Asking researchers to now plan on missing data, and then adopt missing data methods, will be hard. We can re‐assure readers that missing data practices are now very well established (Graham et al., 2006; Nakagawa, 2017) and are rather painlessly implemented in many commonly used statistical software such as *R*, *SAS*, *SPSS* and *MPlus* (see Table 1 for an overview). In fact, many techniques are implemented by default when missing data are included as response variables in models for a number of mixed modelling packages (e.g., model‐based procedures in ‘*MCMCglmm*’ and ‘*ASReml*’). While statistical algorithms vary across these platforms, fairly sophisticated and versatile ones are now implemented in packages for some of the most widely used platforms (e.g., ‘*mice*’, ‘*mi*’, ‘*multimput*’ and ‘*Amelia*’ in the R environment—Table 1) and are under active development (e.g., the *mice* package in R). Statistical procedures for missing data are still rarely taught in undergraduate‐ and graduate‐level courses, so part of the solution will be to begin educating students and practitioners about how to perform statistical procedures to deal with incomplete data, explicitly highlighting some of the challenges and caveats that need to be considered. Nonetheless, there are now excellent resources for learning these methods (Enders, 2010; Gelman & Hill, 2002; Graham, 2009; Nakagawa, 2017; Schafer & Graham, 2002; Su et al., 2011; van Buuren & Groothuis‐Oudshoorn, 2011), and we provide a few case studies in the supplement to show how they can be applied to multilevel data.

### 6.4 Uncertainties surrounding the best PMDD

A big challenge in implementing PMDD is the uncertainty around what the most appropriate missing data design is for a given experiment. This is particularly true in ecology and evolutionary biology because different questions, experimental systems, data structure and measurement variables will require different PMDDs. As such, it will likely be important to test the robustness and power of any given PMDD through simulations (Rhemtulla & Hancock, 2016). With some very simple simulated data, based on effect sizes and experimental designs relevant to the question at hand, the power of different PMDDs can be thoroughly tested during the design stage of an experiment (Enders, 2010). This will require researchers to think carefully about the model they wish to fit to their data so that simulations are realistic for their situation (Rhemtulla & Hancock, 2016). Enders (2010, p. 30) provide a nice introduction on how to conduct power analysis with PMDDs using simulations. While we provide R code showing a few simulated examples and how to apply missing data procedures (see in Appendix S1 at https://doi.org/10.17605/OSF.IO/YZHRN), new multilevel simulation packages, such as SQuID (Allegue et al., 2017) or *simsem* (Pornprasertmanit et al., 2021), allow for researchers to simulate hierarchical data easily across a diversity of design situations. Data can be downloaded and missing data introduced to evaluate the power of different PMDDs.

## 7 CONCLUSIONS AND FUTURE DIRECTIONS

Our goal was to introduce planned missing data designs, along with missing data methods, to ecologists and evolutionary biologists. We have discussed possible missing data designs that can be implemented in research programs and provide case studies along with code to show how missing data procedures can be applied to incomplete hierarchical/multilevel data. While it is still unclear when missing data procedures and PMDD will work best, new statistical methods and a growing awareness of PMDD will likely elucidate answers to these questions (Audigier & Resche‐Rigon, 2017; Audigier et al., 2017; Drechsler, 2015; Quartagno & Carpenter, 2016). We encourage colleagues to begin thinking about PMDDs during experimental design stages to improve research quality and animal welfare while also promoting integrative, cost‐effective research projects in ecology and evolutionary biology.

## CONFLICT OF INTEREST

The authors declare no conflicts of interest.

## Supporting information

Appendix S1

## Data Availability

The data and supplementary materials that support the findings of this study are openly available in the Open Science Framework at https://doi.org/10.17605/OSF.IO/YZHRN.
