# Supplementary material for: Planned missing data designs and methods: Options for strengthening inference, increasing research efficiency and improving animal welfare in ecological and evolutionary research
Source: Evol Appl. 2021 Jul 22;14(8):1958–68. doi: 10.1111/eva.13273 (PMC8372070; doi:10.1111/eva.13273)
Supplement: Supplementary file 1 — Appendix S1 [file EVA-14-1958-s001.html]

Missing data methods and planned missing data designs: Examples of implementaing missing data procedures in three multilevel contexts


Code 

- Show All Code
- Hide All Code

# Missing data methods and planned missing data designs: Examples of implementaing missing data procedures in three multilevel contexts

#### Daniel W.A. Noble and Shinichi Nakagawa

#### 2021-07-20

**Citation**
This supplementary file complements our manuscript on planned missing data designs in ecology and evolution. The manuscript and code associated with it can be cited as follows:

Daniel W.A. Noble & Shinichi Nakagawa. 2021. Planned missing data designs and methods: options for strengthening inference, increasing research efficiency and improving animal welfare in ecological and evolutionary research. Evolutionary Applications. https://doi.org/10.1111/eva.13273.

# 1 General Introduction

In the first two parts of our supplement (Case Studies 1 and 2), we will introduce readers to missing data procedures and how they can be applied to multilevel data using a few different simulated datasets revolving around questions that are commonly encountered in ecological and evolutionary research. These case studies can be thought of as simple versions of planned missing data designs (PMDDs) where missingness is random. Importantly, missing data methods applied to simple missingness scenarios, such as the ones we provide, will give nearly identical results to a complete case analysis providing sample sizes are large, particularly given that we have only simulated missing data in the response (outcome) variable (van Buuren, 2018). It is already well known that multilevel models perform quite well with highly unbalanced data so long as missing data is ‘missing completely at random’ (MCAR) (van Buuren, 2018). In practice, however, missing data will be missing not at random (MNAR), and in both response variables and predictors - possibly even at multiple levels (in a multilevel context) - which can cause major biases and loss of power when applying complete case analyses (Enders, Mistler, & Keller, 2016; Rhemtulla, Jia, Wu, & Little, 2014; van Buuren, 2018).

In our last case study (Case Study 3), in addition to showing how to apply model-based (MB) missing data approaches with multi-response models (when you have more than one response variable), we also conduct simulations that vary sample sizes and levels of missing data to better understand how missing data procedures can help improve the estimation of between-trait correlations with these models. Estimating among-individual correlations between traits is of major interest in many fields of research, including the burgeoning field of animal personality (Dingemanse & Dochtermann, 2013; Réale et al., 2010; Stamps & Groothuis, 2010).

Our intention with these simulations is ***not*** to provide a thorough quantitative assessment on when missing data procedures will be better or worse than standard complete case analyses or what PMDDs will be better over others. Such an assessment will need to be tailored to the different question(s) researchers seek to answer and their study systems. We merely want to demonstrate how MB and multiple imputation (MI) procedures can be applied to multilevel data with different error structures and under different contexts. As such, readers should not assume that the application of missing data procedures, or the results from our simulation, will be generalisable to all situations. For more thorough simulation studies, particularly in the context of mixed models and understanding the power of different PMDDs, we refer readers to the many excellent simulation studies that already exist (Enders, Mistler, & Keller, 2016; Lüdtke Grund S. & Robitzsch, 2016; L¨udtke Grund S. & Robitzsch, 2018; S. Grund, Lüdtke, & Robitzsch, 2017; Lüdtke, Robitzsch, & Grund, 2017; Resche-Rigon & White, 2018; Rhemtulla, Jia, Wu, & Little, 2014).

Throughout, R code is presented to help the reader understand the simulations along with example analysis code. For Case Studies 1 & 2, we show multiple packages and equivalent approaches researchers can use with the same data to give flexibility in the inferential and missing data methods they may want to use. Importantly, the code is meant to help readers get familiar with applying missing data procedures to different situations. We recommend that readers always read and understand the documentation and methods when applying these models to their particular data.

# 2 Packages

There are a few packages that you will need to download and load before running the simulations. For all relevant code chunks, the code can be ‘copied’ by clicking the ‘copy’ button in the top right corner of the box.

```
# install.packages('pacman') # If you don't have pacman installed,
# please do so first. Remove the '#'.

pacman::p_load(plyr, asreml, akima, fields, viridis, VIM, lme4, mice, miceadds,
    MASS, png, knitr, kableExtra, brms, rstan, magick, patchwork, tidyverse,
    broom.mixed)
```

# 3 Case Study 1: Model-based approaches to handle missing data in simulated multilevel linear growth models

## 3.1 Introduction

To show how one might make use of model based missing data methods for a simple PMDD, when both fixed and random effects might be of interest, we present a case study where we have manipulated the early thermal environment of a sample of lizard eggs – a common approach in lizard research (e.g., Noble, Stenhouse, & Schwanz, 2018). Here, we are interested in understanding how the thermal environment lizards experience affect the growth curves of animals within each treatment. We will simulate some data assuming that individuals follow a linear growth trajectory (random regression model), at least over the period in which we measured their weight, according to the following model:

\[m\_{ij} = (\beta\_{0} + \alpha\_{i}) + \beta\_{1}T\_{ij} + (\beta\_{2} + s\_{i})A\_{ij} + \beta\_{3}(T\_{ij} \* A\_{ij}) + \epsilon\_{ij}\]

where \(m\_{ij}\) is the mass of individual *i* for observation *j*, \(T\_{ij}\) is a dummy variable (‘0’ or ‘1’) indicating whether observation *j* for individual *i* belongs to the control group (23°C) or the treatment group (26°C), \(A\_{ij}\) is the age of individual *i* at observation *j*, \(\beta\_{1}\) is the contrast between the control group mean at age 0 (\(\beta\_{0}\)) and the treatment mean at age 0, \(\beta\_{2}\) is the effect of age on mass, and \(\beta\_{3}\) is the difference in mass change across age between treatments (i.e., interaction effect). \(\alpha\_{i}\) and \(s\_{i}\) are individual level random effects for the random intercept and slope, respectively, which are assumed to follow an ~\(MVN([0,0],[0.5,0.25,0.25,0.4])\) , and \(\epsilon\_{ij}\) is the observation-level random effect assumed to follow an ~\(N(0,1)\). In this case study, we will also assume that lizards not reaching a certain mass at day 1 die early, and are missing completely from the data set to add a bit of biological reality to missing data.

## 3.2 Simulated data

These data can be simulated as follows:

```
# Grab our functions
source("./func.R")

# Parameters
sims = 1500  # Number of simulated datasets
k1 = 20  # Number of individuals #20
n = 10  # Number of observations per individual #10
b0 = 1.2  # Intercept
b1 = 4  # Treatment contrast
b2 = 1.8  # effect of age
b3 = 1.6  # Interaction

# Set seed
set.seed(6798)

# Simulate a growth model
age <- rep(seq(1, n), k1)
trt <- rep(c(0, 1), each = (k1 * n)/2)
ind <- as.character(rep(1:k1, each = n))

# Random effects
SI <- matrix(c(0.5, 0.25, 0.25, 0.4), nrow = 2, ncol = 2)
re <- data.frame(MASS::mvrnorm(k1, c(0, 0), SI))
re_ind <- re[rep(seq_len(nrow(re)), each = n), ]

# Assume linear growth
M = (b0 + re_ind[, 1]) + b1 * trt + (b2 + re_ind[, 2]) * age + b3 * (age * trt) +
    rnorm(k1 * n, 0, 1)

# Create data frame
data_complete <- data.frame(ind = as.factor(ind), trt = trt, age, M)

# Create a real case scenerio and say any animal under 4 grams at age 1 dies,
# so we have no growth data on them. In this case we have some MNAR data, but
# will be MAR when imputing

splt <- split(data_complete, data_complete$ind)

dead <- function(x) {
    if (x$M <= 4 & x$age == 1) {
        x$dead <- 1
    } else {
        x$dead <- 0
    }
    return(x)
}

data_complete_mort <- plyr::ldply(lapply(splt, function(x) dead(x)))

# Create missing data. Here we want to see if missing 30%, which mean the
# actual sample size is the same as part I
data_missing <- createMiss(data_complete_mort, grp = "ind", missVar = "M", p.miss = 0.3,
    type = "w")

# Missing data based on mass and age
data_missing$M <- ifelse(data_missing$dead == 1, NA, data_missing$M)
```

We can see what the simulated data look like (Figure 3.1). Notice that we are missing data within individuals across age and we have fewer individuals in the cold treatment because they are a smaller mass and therefore have compromised survival.

Figure 3.1: Simulated experimental data on growth rates across age for lizards that were incubated at 23°C (cold) and another sample incubated at 26°C (hot). There is lower survival of animals from the cold treatment compared to the hot treatment and so we are missing more data on lizards in the cold treatment.

## 3.3 Applying model-based missing data methods to incomplete data

Now that we have simulated data we can use model-based missing data approaches to automatically deal with the incomplete data when fitting the model. While there are a number of packages that can be used to do this, we focus on providing code for comparable frequentist (`ASReml-R`) and Bayesian models (`MCMCglmm` or `brms`). We simply want to provide users with an understanding of how they can fit incomplete data in a multilevel context and show that, even if it is may be less efficient at times, it will recover the correct answers.

```
## Frequentist Approach: Models fit with ASREML-R

# Model that uses the incomplete data
modASREML_data_missing <- asreml::asreml(fixed = M ~ trt + age + trt:age, random = ~str(~ind +
    age:ind, ~us(2, init = c(1, 0.1, 1)):id(20)), data = data_missing, maxiter = 20,
    na.action = asreml::na.method(y = "include"))
```

```
## Online License checked out Tue Jul 20 18:50:52 2021
## Model fitted using the gamma parameterization.
## ASReml 4.1.0 Tue Jul 20 18:50:52 2021
##           LogLik        Sigma2     DF     wall    cpu
##  1      -89.1681       1.01124     87 18:50:52    0.0 (1 restrained)
##  2      -87.1038       1.33660     87 18:50:52    0.0
##  3      -85.5847       1.25739     87 18:50:52    0.0
##  4      -84.8996       1.20056     87 18:50:52    0.0
##  5      -84.7369       1.16833     87 18:50:52    0.0
##  6      -84.7317       1.16242     87 18:50:52    0.0
```

```
fe <- round(summary(modASREML_data_missing, coef = TRUE)$coef.fixed, 3)
re <- round(summary(modASREML_data_missing)$varcomp[, c(1:3)], 3)

list(Random_Effects = re, Fixed_Effects = fe)
```

```
## $Random_Effects
##                       component std.error z.ratio
## ind+age:ind!us(2)_1:1     0.733     0.691   1.061
## ind+age:ind!us(2)_2:1     0.139     0.176   0.789
## ind+age:ind!us(2)_2:2     0.204     0.096   2.123
## units!R                   1.162     0.204   5.693
## 
## $Fixed_Effects
##             solution std error z.ratio
## trt:age        1.208     0.311   3.886
## age            1.820     0.272   6.681
## trt            3.658     0.807   4.532
## (Intercept)    1.898     0.704   2.695
```

We can see the model fits above. Both the random effects estimates and the fixed effect estimates and their uncertainty capture our true simulated coefficients. `ASReml-R` is fast, but not free, unfortunately. We can also implement model-based approaches using Bayesian models quite easily. We’ll show how using two different packages below:

```
## Bayesian Approach: Models fit with MCMCglmm
         
  # For Bayeisan models, we need to set the prior. Given our data is simple, we will use the default priors for fixed effects and specify priors for random effects. 

     prior <- list(R = list(V = 1, nu =0.001),                         # Prior on residual variance
                       G = list(G1=list(V=diag(2),                         # Prior for random slope and intercept
                                        nu = 2, 
                                        alpha.mu = rep(0,2), 
                                        alpha.V = diag(25^2,2,2))))
        
  # Samples to take from the posterior distribution
        nitt = 220000
      burnin = 20000
        thin = 50
        
  # Model that uses the incomplete data
        modMCMCglmm_data_missing <- MCMCglmm::MCMCglmm(M ~ trt + age + trt:age, 
                                            random = ~us(1 + age):ind, 
                                            data = data_missing, 
                                            prior = prior, 
                                            nitt = nitt, burnin = burnin,
                                            thin = thin, verbose = FALSE)
        
        fe_MCMC <- round(summary(modMCMCglmm_data_missing)$solutions, 3)[,c(1:3,5)]
        re_MCMC <- round(summary(modMCMCglmm_data_missing)$Gcovariances, 3)[,c(1:3)]
        list(Random_Effects = re_MCMC, Fixed_Effects = fe_MCMC)
```

```
## $Random_Effects
##                             post.mean l-95% CI u-95% CI
## (Intercept):(Intercept).ind     1.179    0.000    3.487
## age:(Intercept).ind             0.092   -0.388    0.595
## (Intercept):age.ind             0.092   -0.388    0.595
## age:age.ind                     0.315    0.076    0.682
## 
## $Fixed_Effects
##             post.mean l-95% CI u-95% CI pMCMC
## (Intercept)     1.896    0.343    3.494 0.024
## trt             3.652    1.897    5.581 0.001
## age             1.820    1.122    2.514 0.000
## trt:age         1.210    0.413    1.970 0.006
```

If users are not totally comfortable with `MCMCglmm` the package `brms` also provides very user friendly functions to help with model-based imputation and the code can be written in a similar style to the very popular `lme4` package. We’ll show how we can impute the same data above.

```
model_brms <- bf(M | mi() ~ trt + age + trt:age + (1 + age | ind))
model_brms_fit <- brm(model_brms, data = data_missing, chains = 4, iter = 6000, thin = 10,
    open_progress = FALSE, refresh = 0)
summary(model_brms_fit)
```

```
##  Family: gaussian 
##   Links: mu = identity; sigma = identity 
## Formula: M | mi() ~ trt + age + trt:age + (1 + age | ind) 
##    Data: data_missing (Number of observations: 200) 
## Samples: 4 chains, each with iter = 6000; warmup = 3000; thin = 10;
##          total post-warmup samples = 1200
## 
## Group-Level Effects: 
## ~ind (Number of levels: 20) 
##                    Estimate Est.Error l-95% CI u-95% CI Rhat Bulk_ESS Tail_ESS
## sd(Intercept)          0.96      0.52     0.08     2.10 1.00     1080      986
## sd(age)                0.54      0.15     0.32     0.93 1.00     1009      824
## cor(Intercept,age)     0.27      0.41    -0.59     0.92 1.00     1016      992
## 
## Population-Level Effects: 
##           Estimate Est.Error l-95% CI u-95% CI Rhat Bulk_ESS Tail_ESS
## Intercept     1.93      0.80     0.36     3.57 1.00     1215     1139
## trt           3.63      0.90     1.87     5.42 1.00     1234     1115
## age           1.81      0.33     1.19     2.49 1.00      703      800
## trt:age       1.23      0.38     0.45     1.95 1.00      800      840
## 
## Family Specific Parameters: 
##       Estimate Est.Error l-95% CI u-95% CI Rhat Bulk_ESS Tail_ESS
## sigma     1.11      0.10     0.94     1.32 1.00     1027     1200
## 
## Samples were drawn using sampling(NUTS). For each parameter, Bulk_ESS
## and Tail_ESS are effective sample size measures, and Rhat is the potential
## scale reduction factor on split chains (at convergence, Rhat = 1).
```

Bayesian approaches offer a lot of flexibility and resolve some of the limitations of model-based approaches described in Case Study 2 under a likelihood framework (e.g., estimations of uncertainty for variance components; see below). Of course, if we were able to get complete data, this will always be the best. While a complete case analysis in this simple simulated case will give you essentially the same answer as the model-based missing data approach, this will likely not be the case in real data, with more severe consequences in a multilevel context (van Buuren, 2018). Often unintentional missing data will be common, and multiple variables will be missing data. Missing data, could in fact be missing not at random (MNAR) and can therefore bias parameter estimates and impact inferential statistics, sometimes in very severe ways. The benefit of using a model-based approach is that it will give you the same result as a complete case analysis if missing data is random, as is the case here, but will also help deal with any missing data in the response that is missing not at random.

# 4 Case Study 2: Multiple imputation approaches to handle missing multilevel count data (Poisson error distribution)

## 4.1 Introduction

The use of generalised linear mixed models (GLMMs) in ecology and evolution has exploded in recent times. We often collect data that is not normally distributed, and so, rely on different families of error distributions to more effectively model these types of data. Model-based (MB) approaches can be very powerful, but they do come with certain limitations. First, because we fit a single model with incomplete data all the variables we need to help with imputation also need to be included in the model. That’s fine if you have a simple problem but often we have a suite of variables that are useful for dealing with missing data, but that we don’t want to include in our model because of the greater complexity in model interpretation that comes with estimating additional parameters. This will be the case if you have auxiliary variables that are not of biological interest, but that are likely going to help ensure your data are missing at random (MAR). Second, currently many model-based implementations assume multivariate normality. For many problems this may be fine, but often with multilevel data we are modelling variables that have non-normal error distributions, such as when we have count or proportion (binomial) data. Finally, we are often not just missing data in our response variable, but also in predictor variables, which MI can help deal with quite well. Multiple imputation can be quite helpful in providing more flexible options to users to get around these limitations. It is important to note that both MI and MB missing data procedures perform equally well for similar types of problems, and so, when one would use MI over MB just comes down to the context and preference.

## 4.2 Simulated data

To re-assure readers that missing data procedures can, and do, work with non-normal distributions assume we are interested in provisioning rates (the number of feeding visits by a parent) in a bird species, the fictitious Missing Capped Warbler (*Sylvia absenscapilla*) (Figure 4.1). We would like to understand the costs of female provisioning by experimentally manipulating brood sizes (n = 6 chicks) in a random sample of birds compared to a control group, which has normal brood sizes (n = 3 chicks) (Liebl, Browning, & Russell, 2016). The Missing Capped Warbler is notoriously difficult to observe as it is found in thick scrub, and so, we placed cameras at random nests during the first two weeks of the breeding season to observe provisioning rates in the two treatments over a 5-hour period. Provisioning rates are known to change as chicks develop (Khwaja et al., 2017), and so, the cameras were on each nest for a total of 20 days to understand how the demands of chicks change, and whether females can keep up with these demands. While it is easy to capture data with cameras, unfortunately, it is a laborious process to observe all the resulting video for 40 birds measured over 20 days (a total of 4000 hours of video!). We therefore decided to implement a PMDD, where we randomly sampled a set of videos (n = 536 out of 800 videos; ~ 33% missing data) where provisioning rates can be quantified (cutting the total hours of video watching to 2680 hours).

Here, our data (number of visits in 5 hours) is Poisson distributed, and hierarchical in nature (individual females vary in their responses). We can use the `mice` package (van Buuren & Groothuis-Oudshoorn, 2011) to impute both planned and any unplanned (e.g. camera failures) missing data. Provisions were simulated assuming a Poisson error distribution using the following model: \(n\_{ji}= (\beta\_{0} + \alpha\_{i}) + \beta\_{1}T\_{ji} + (\beta\_{2} + s\_{i})A\_{ji} + \epsilon\_{ji}\) where Provisions = log(\(n\_{ji}\)). \(\beta\_{0}\) = 2; \(\beta\_{1}\)= 1; \(\beta\_{2}\)= 0.01 with a random intercept and slope variance and covariance matrix as: ~\(MVN([(0,0)],[(0.01, 0, 0, 0.0025)])\).

We can simulate these data as follows:

```
# Parameters
set.seed(7)  # Set seed to reproduce simulated data
k = 40  # Number of individuals 
n = 20  # Number of trials or repeated measurements for each individual 
trtE = 1  # Treatment effect we will model. Brood size
ageE = 0.01  # Often there age effects for provisioning
bo = 2  # Intercept


# Dummy variable for treatment
trt <- rep(c(1, 0), each = (k * n)/2)

# Number of trials for each individual
age <- rep(1:n, k)
SI <- matrix(c(0.01, 0, 0, 0.0025), nrow = 2, ncol = 2)

# Random effects.
re <- data.frame(MASS::mvrnorm(k, c(0, 0), SI))

# Replicate these effects to expand to number of individual observations
re_ind <- re[rep(seq_len(nrow(re)), each = n), ]

# ID identifier
ind <- as.character(rep(1:k, each = n))

# Create a dataframe with the variables
data <- data.frame(ind, age, trt)
data$trt_name <- ifelse(data$trt == 0, "Control", "Experimental")

# Simulate some provisioning data based on the probabilities for each
# observation.
data$provision <- rpois(k * n, exp((bo + re_ind[, 1]) + (trtE) * trt + (ageE + re_ind[,
    2]) * age))

# Now lets assume 33% of our data is missing randomly for each individual
data <- createMiss(data, grp = "ind", missVar = "provision", p.miss = 0.33, type = "w")
```

Now that we have simulated some data we can have a look at what it looks like. Figure 4.1 shows how provisioning rate changes across age in the two experimental groups (‘Control’ and ‘Experimental’), and we see higher provisioning in the experimental treatment group compared to the control, on average. However, there is a lot of individual variation.

Figure 4.1: Example data showing the provisioning rates (number of feeding visits within a 5-hour period) for control (3 eggs) and experimentally elevated (6 eggs) brood sizes in the fictitious Missing Capped Warbler across the first 20 days of chick age.

## 4.3 Applying multiple imputation methods to missing multilevel count data

We now have our incomplete data for which we can use multiple imputation to impute plausible missing values for incidences where we are missing data and add some variation to the imputed values. We will do this a total of 20 times to get 20 complete data sets for which we can then estimate our parameters of interest.

```
# We need to set the class variable, or random effect grouping variable as an
# integer. Also, we can do a quick mice run to generate the predictor matrix,
# which we can then easily modify for the proper run.

data$ind <- as.integer(data$ind)
imp <- mice(data, maxint = 0, printFlag = FALSE)
pred <- imp$pred

# For multilevel data we need to specify the class variable (i.e. individual)
# using a -2 (this variable needs to be integers). The 2 indicates that the
# variable is considered as both a fixed and random effect. 1 indicates that
# this variable should only be modelled as a fixed effect.
pred["provision", ] <- c(-2, 2, 1, 0, 0)
pred[, "provision"] <- c(-2, 2, 1, 0, 0)

# Now lets do a proper imputation setting the 2-level approach for
# provisioning. We'll impute m = 20 imputed, complete datasets.

imp <- mice(data, m = 20, meth = "2l.pmm", pred = pred, printFlag = FALSE)

fit <- with(imp, lme4::glmer(provision ~ trt + age + (1 + age | ind), family = "poisson"))
summary(pool(fit))
```

```
##          term    estimate   std.error  statistic        df   p.value
## 1 (Intercept) 2.115511426 0.077404002 27.3307758  91.21209 0.0000000
## 2         trt 0.873687574 0.076037143 11.4902736 432.57596 0.0000000
## 3         age 0.006046951 0.008298938  0.7286415 242.44508 0.4669242
```

We will use a 2 level predictive mean matching method (`2l.pmm`) using the `mice` and `miceadds` packages. Predictive mean matching doesn’t assume the variable being imputed is normally distributed, and deals with continuous variables. If you had binary or proportion data, then `2l.binary` can be used as the method of choice instead. In this case, we see that imputing the data gets us remarkably close to our true simulated parameters. Further, if you have missing values at the level 2 (the clustering level) rather than level 1 (the observation level), they can be imputed by similar methods such as `2lonly.pmm`. This is quite important because missing data at higher levels can case severe biases in model parameters and uncertainty when implementing a complete case analysis (van Buuren, 2018).

Non-normal data can also be imputed using MB approaches, but this is most easily applied in a Bayesian setting. In this case it works because we only having missing data in our response variable. However, if we also had missing data in our predictors multiple imputation would be needed. To show users how they can apply model-based approaches to these simulated data we’ll turn again to `MCMCglmm` which provides excellent facilities to model different response distributions.

```
prior <- list(R = list(V = 1, nu = 0.001), G = list(G1 = list(V = diag(2), nu = 0.002)))
nitt = 520000
burnin = 20000
thin = 100

modMCMCglmm_data_miss <- MCMCglmm::MCMCglmm(provision ~ trt + age, random = ~us(1 +
    age):ind, data = data, prior = prior, nitt = nitt, burnin = burnin, thin = thin,
    family = "poisson", verbose = FALSE)
summary(modMCMCglmm_data_miss)
```

```
## 
##  Iterations = 20001:519901
##  Thinning interval  = 100
##  Sample size  = 5000 
## 
##  DIC: 3029.629 
## 
##  G-structure:  ~us(1 + age):ind
## 
##                             post.mean   l-95% CI u-95% CI eff.samp
## (Intercept):(Intercept).ind  0.008851  0.0003782 0.021418    498.2
## age:(Intercept).ind          0.001338 -0.0014327 0.004230    646.8
## (Intercept):age.ind          0.001338 -0.0014327 0.004230    646.8
## age:age.ind                  0.002271  0.0012843 0.003475   1777.3
## 
##  R-structure:  ~units
## 
##       post.mean  l-95% CI u-95% CI eff.samp
## units  0.001341 9.401e-05  0.00389    346.8
## 
##  Location effects: provision ~ trt + age 
## 
##             post.mean  l-95% CI  u-95% CI eff.samp  pMCMC    
## (Intercept)  1.953340  1.862328  2.048313    608.7 <2e-04 ***
## trt          0.986419  0.871451  1.103943    549.6 <2e-04 ***
## age          0.007675 -0.007365  0.023145   3322.7  0.324    
## ---
## Signif. codes:  0 '***' 0.001 '**' 0.01 '*' 0.05 '.' 0.1 ' ' 1
```

While there are some problems with the MCMC chains mixing, this gives us exactly the same answers as the multiple imputation results. Running the model longer or changing the priors may help improve MCMC chain mixing.

# 5 Case Study 3: Model-based simulation with a multi-response model to estimate between-individual correlation between two traits with missing data

## 5.1 Introduction

Many research fields (e.g, quantitative genetics, behavioural ecology) are interested in using variance partitioning methods to quantify within and between group (e.g., between individual) (co)variances among a set of traits (Dingemanse & Dochtermann, 2013). For example, the field of animal personality is interested in behavioural syndromes, or consistent between individual correlations among behavioural traits (Réale et al., 2010; Sih, Bell, Johnson, & Ziemba, 2004; Stamps & Groothuis, 2010). The presence of strong between individual correlations implies behavioural traits are somewhat constrained. Here we will simulate some simple data to demonstrate how MB methods can be easily adopted to estimate among individual correlations between two traits (e.g., \(Y\_{1}\) and \(Y\_{2}\)) in a hierarchical multi-response model. We also use this example to do a very simple simulation to show how it can help improve standard errors in this particular situation. The model we wish to simulate here is as follows:

\[ \mathbf{Y\_{jik}= α\_{k} + b\_{ik} + w\_{jik}} \]

\[ \begin{bmatrix}
Y\_{ij1}\\
Y\_{ij2}\\
Y\_{ij3}
\end{bmatrix} \sim \left ( \begin{bmatrix}
2\\
4\\
10
\end{bmatrix}, \mathbf{B} + \mathbf{W} \right )\]

\[ \mathbf{B} = \begin{pmatrix}
1&0.4 & -0.05\\
0.4 & 1 & -0.40\\
-0.05 & -0.40 & 1
\end{pmatrix}\]

\[ \mathbf{W} = \begin{pmatrix}
1 & 0 & 0\\
0 & 1 & 0\\
0 & 0 & 1
\end{pmatrix}\]

Here, \(Y\_{jik}\) is a matrix containing *j* observations, for each individual, *i*, for the *k* traits (\(Y\_{1}, Y\_{2}, Y\_{3}\)); \(α\_{k}\) is a scalar vector containing the intercept for each trait \([2,4,10]^T\) (means for \(Y\_{1} – Y\_{3}\)); \(b\_{ik}\) is a matrix containing the individual specific random effects or deviations for individual, *i* , for each trait, *k*, with \(b\_{ik}\) ~ \(MVN([0,0,0]^T,\mathbf{B})\). \(w\_{jik}\) is the *j* observation level deviation for each individual *i* for the three traits *k* with \(w\_{jik}\) ~ \(MVN([0,0,0]^T,\mathbf{W})\). Note here that \(Y\_{3}\) is not of interest with respect to the question, but because it is correlated with \(Y\_{2}\) it could be used as an auxiliary variable to improve the parameter estimation process.

In this example, we will miss data completely randomly throughout the variables \(Y\_{1} - Y\_{3}\). This is the simplest planned missing data design (PMDD) that one can implement. As indicated above, while mixed models normally can handle missing data well with randomly missing data in the response variable, in this case we have missing data in multiple response variables. As such, whether we use a complete case or model-based approach will make a difference in our ability to estimate the uncertainty around between-individual correlations, as we will see.

## 5.2 Simulated data and small simulation

To begin we need to define a set of parameters to run this simulation. These included, *k*, the number of individuals measured; *n*, the total number of times each individual was measured and the percentage of missing data, ranging from 5-50% (defined in the ‘missing’ vector). Defining these in R is as follows:

```
k = c(100, 80, 60, 40, 30, 15, 10)  # Number of individuals
n = 10  # Number of times each individual measured
sims = 5000  # Number of simulations
missing = c(0.05, 0.1, 0.2, 0.3, 0.4, 0.5)  # Percentage of missing data
```

Once our parameters are defined we can then create our between and within-individual co-variance matrices which will allow us to simulate data under known situations. To do this, we first need to define the correlations and standard deviations for the variables were are interested in:

```
# Between individual correlations (cor) between traits (Y_{1}, Y_{2} and Y_{3},
# which equals 1,2,3, respectively) and their corresponding standard deviations
# (SD_).
corY1Y2 = 0.4
corY1Y3 = -0.05
corY2Y3 = -0.4

SD_Y1 = 1
SD_Y2 = 1
SD_Y3 = 1

# Within individual correlations (cor) between traits (A, B and C, which equals
# 1,2,3, respectively) and their corresponding standard deviations (SD).
corWY1Y2 = 0
corWY1Y3 = 0
corWY2Y3 = 0

WSD_Y1 = 1
WSD_Y2 = 1
WSD_Y3 = 1
```

Now that we have defined the correlations and standard deviations between our traits we can construct our co-variance matrices. Remember that the variance (V) and covariance (cov) are defined as follows:
\[V\_{1} = SD^2\_{1}\]
\[cov\_{1,2} = r\_{1,2}SD\_{1}SD\_{2}\]

Therefore, from our parameters we can define the covariance matrices as follows:

```
# Between-individual co-variance matrices for three traits.
B <- matrix(c((SD_Y1^2), corY1Y2 * SD_Y1 * SD_Y2, corY1Y3 * SD_Y1 * SD_Y3, corY1Y2 *
    SD_Y1 * SD_Y2, (SD_Y2^2), corY2Y3 * SD_Y2 * SD_Y3, corY1Y3 * SD_Y1 * SD_Y3, corY2Y3 *
    SD_Y2 * SD_Y3, (SD_Y3^2)), nrow = 3, ncol = 3, byrow = TRUE)


# Within-individual co-variance matrices for three traits.
W <- matrix(c((WSD_Y1^2), corWY1Y2 * WSD_Y1 * WSD_Y2, corWY1Y3 * WSD_Y1 * WSD_Y3,
    corWY1Y2 * WSD_Y1 * WSD_Y2, (WSD_Y2^2), corWY2Y3 * WSD_Y2 * WSD_Y3, corWY1Y3 *
        WSD_Y1 * WSD_Y3, corWY2Y3 * WSD_Y2 * WSD_Y3, (WSD_Y3^2)), nrow = 3, ncol = 3,
    byrow = TRUE)
```

This then gives us the between individual matrix:

```
B
```

```
##       [,1] [,2]  [,3]
## [1,]  1.00  0.4 -0.05
## [2,]  0.40  1.0 -0.40
## [3,] -0.05 -0.4  1.00
```

And the within-individual matrix:

```
W
```

```
##      [,1] [,2] [,3]
## [1,]    1    0    0
## [2,]    0    1    0
## [3,]    0    0    1
```

We can now use these to simulate our multilevel data according to the above model. We’ll demonstrate with just one value of k (100) as follow:

```
individual <- gl(k[1], n)
Bmu = c(0, 0, 0)
Wmu = c(2, 4, 10)
rB <- as.data.frame(MASS::mvrnorm(k[1], mu = Bmu, B))
rW <- as.data.frame(MASS::mvrnorm(k[1] * n, mu = Wmu, W))

y <- rB[individual, ] + rW
data <- data.frame(y, individual)
colnames(data) <- c("y1", "y2", "y3", "ID")
```

Now that we have our functions defined we can run our simulations looping through the different parameter combinations. There are many ways to simplify this process and it could even be wrapped into a function itself, but it is often (if not taking too long) easier to visualize and run these simulations with loops.

```
# Summarise the true, imputed and missing data results
muTrue <- list()
muImpute <- list()
muMiss <- list()

set.seed(78)
system.time(for (i in 1:length(k)) {
    for (j in 1:length(missing)) {
        miss <- rep(missing[j], each = 3)

        # Create full and missing data sets
        simData <- datSim(k = k[i], n = n, sims = sims, B = B, W = W)
        missData <- lapply(simData, function(x) createMiss(x, grp = "ID", p.miss = miss,
            type = "all"))

        # Extract estimates from each dataset
        simsTrue <- lapply(simData, function(x) asremlEst(x))
        simsImpute <- lapply(missData, function(x) asremlEst(x, na.method.Y = "include"))
        missDataCC <- lapply(missData, function(x) x[complete.cases(x), ])
        simsMiss <- lapply(missDataCC, function(x) asremlEst(x))

        # Once estimates are extracted add these into a growing list
        muTrue[[paste(k[i] * n, missing[j] * 100)]] <- EstSum(simsTrue)
        muImpute[[paste(k[i] * n, missing[j] * 100)]] <- EstSum(simsImpute)
        muMiss[[paste(k[i] * n, missing[j] * 100)]] <- EstSum(simsMiss)
    }
})

simResults <- list(muTrue = muTrue, muImpute = muImpute, muMiss = muMiss)
```

This code chunk will run through each parameter value of k and then, at this level of k, generate 5000 data sets and apply each level of missingness in *missing* until it completes all elements of the *missing* object. As it does this it will collect data on parameter estimates from the models fit to each of the 5000 data sets and summaries these in the empty vectors. The workhorse for this is the `datSim` function in the `.func.R` script. It will also record how many models have had convergence problems. Once all this is complete it will repeat this entire process at the new level of k, continuing through the elements of k until it has cycled through them all. From this simulation you will get parameter estimates (in this case for the correlation between Y1:Y2) across three different contexts: 1) The complete simulated data; 2) The missing data set that has had values imputed through data augmentation and 3) The missing data set where a complete case approach has been used. Note that this makes use of the following `ASReml` model:

```
# Multi-response model fit with Asreml-R
mod_asreml <- asreml(cbind(y1, y2, y3) ~ trait - 1, random = ~us(trait, init = rep(0,
    6)):ID, residual = ~units:us(trait, init = rep(0, 6)), data = data, maxiter = 20)
```

`ASReml-R` was used here given it is quite fast and it can run these simulations efficiently. However, an equivalent model that can be fit using `MCMCglmm` is as follows:

```
# Multi-response model fit with MCMCglmm
mod_MCMCglmm <- MCMCglmm(cbind(y1, y2, y3) ~ trait - 1, random = ~us(trait):ID, rcov = ~us(trait):units,
    family = rep("gaussian", 3), data = data, nitt = 220000, burnin = 20000, thin = 100)
```

This model should run fine without the need to set the prior. However, in real situations this will need to be specified. Note that reproducing this simulation will take a long time. We provide the simulated results in the Open Science Framework repository.

## 5.3 Summarizing and Graphing Simulation Results

Looking at a single situation, say a sample size of 300 with 30% missing data, we can compare the standard errors for the between-individual level correlation. In practice, this means we are comparing: 1) N = 300 with 30% missing data where missing measurements are dealt with using a case-wise likelihood approach (FIML) during model fitting to 2) a complete case analysis that uses a total sample size of N = 270. In other words, this means comparing a situation where, possibly due to logistical constraints, data is missed randomly and missing measurements included in the analysis (Figure 5.1A) compared to a second situation where only a random sample of N = 270 data points are collected (Figure 5.1B). Since data is missing randomly here in both cases, and this is a simple situation, the correlation coefficients will be nearly the same.

From the complete-case analysis, we can see that the mean correlation estimated is r = 0.395 with an SE (standard error) = 0.26. Comparing the same situation where we have used MB approaches with incomplete data our estimated correlation is r = 0.397 with an SE = 0.225. When we get to smaller sample sizes, MB approaches have the additional benefit of improving the likelihood of model-convergence with more gains in the SE estimation. For example, if we had tried to collect n = 100 data points, but we were missing n = 40 (40%), lets assume because of random equipment failure, the situation would be different. Complete case analysis leads to a higher probability of the model not converging with about 3% of models not converging and estimating an r = 0.382 with an SE = 0.578. In contrast, the same situation using model-based missing data procedures led to no convergence issues with an improved estimation of r = 0.41 and SE = 0.415.

It is important to note that not all situations we simulate will result in missing data procedures improving upon standard errors. This is an important point to remember. When you have very little missing data or large sample sizes missing data procedures can even be less efficient than complete case analyses when missing measurements are random.

We can view the full parameter space by looking at Figure 5.1.

Figure 5.1: Standard error estimates for the among-individual level trait correlation from 5000 simulations across varying levels of missing data and sample size for A) Full information maximum likelihood model-based approaches (FIML) and B) Complete case analysis

# 6 Functions

The functions used throughout can be sourced by `source("./func.R")` with the provided `func.R` script. The entire supplement, R script and simulation results can also be accessed from the Open Science Framework (OSF) at https://doi.org/10.17605/OSF.IO/YZHRN.

# 7 Session Information

**R version 4.0.3 (2020-10-10)**

**Platform:** x86\_64-apple-darwin17.0 (64-bit)

**locale:**
en\_AU.UTF-8||en\_AU.UTF-8||en\_AU.UTF-8||C||en\_AU.UTF-8||en\_AU.UTF-8

**attached base packages:**
*grid*, *stats*, *graphics*, *grDevices*, *utils*, *datasets*, *methods* and *base*

**other attached packages:**
*broom.mixed(v.0.2.7)*, *forcats(v.0.5.1)*, *stringr(v.1.4.0)*, *dplyr(v.1.0.7)*, *purrr(v.0.3.4)*, *readr(v.1.4.0)*, *tidyr(v.1.1.3)*, *tibble(v.3.1.2)*, *tidyverse(v.1.3.1)*, *patchwork(v.1.1.1)*, *magick(v.2.7.2)*, *rstan(v.2.21.2)*, *ggplot2(v.3.3.5)*, *StanHeaders(v.2.21.0-7)*, *brms(v.2.15.0)*, *Rcpp(v.1.0.7)*, *kableExtra(v.1.3.4)*, *knitr(v.1.33)*, *png(v.0.1-7)*, *MASS(v.7.3-54)*, *miceadds(v.3.11-6)*, *mice(v.3.13.0)*, *lme4(v.1.1-27.1)*, *VIM(v.6.1.0)*, *colorspace(v.2.0-2)*, *fields(v.12.5)*, *viridis(v.0.6.1)*, *viridisLite(v.0.4.0)*, *spam(v.2.7-0)*, *dotCall64(v.1.0-1)*, *akima(v.0.6-2.1)*, *asreml(v.4.1.0.154)*, *Matrix(v.1.3-4)* and *plyr(v.1.8.6)*

**loaded via a namespace (and not attached):**
*pacman(v.0.5.1)*, *utf8(v.1.2.1)*, *tidyselect(v.1.1.1)*, *htmlwidgets(v.1.5.3)*, *ranger(v.0.13.1)*, *munsell(v.0.5.0)*, *codetools(v.0.2-18)*, *DT(v.0.18)*, *miniUI(v.0.1.1.1)*, *withr(v.2.4.2)*, *Brobdingnag(v.1.2-6)*, *highr(v.0.9)*, *rstudioapi(v.0.13)*, *stats4(v.4.0.3)*, *robustbase(v.0.93-8)*, *vcd(v.1.4-8)*, *bayesplot(v.1.8.1)*, *labeling(v.0.4.2)*, *emmeans(v.1.6.2-1)*, *farver(v.2.1.0)*, *bridgesampling(v.1.1-2)*, *coda(v.0.19-4)*, *vctrs(v.0.3.8)*, *generics(v.0.1.0)*, *TH.data(v.1.0-10)*, *xfun(v.0.24)*, *R6(v.2.5.0)*, *markdown(v.1.1)*, *gamm4(v.0.2-6)*, *projpred(v.2.0.2)*, *assertthat(v.0.2.1)*, *promises(v.1.2.0.1)*, *scales(v.1.1.1)*, *multcomp(v.1.4-17)*, *nnet(v.7.3-16)*, *gtable(v.0.3.0)*, *processx(v.3.5.2)*, *sandwich(v.3.0-1)*, *rlang(v.0.4.11)*, *systemfonts(v.1.0.2)*, *splines(v.4.0.3)*, *broom(v.0.7.8)*, *inline(v.0.3.19)*, *yaml(v.2.2.1)*, *reshape2(v.1.4.4)*, *abind(v.1.4-5)*, *modelr(v.0.1.8)*, *threejs(v.0.3.3)*, *crosstalk(v.1.1.1)*, *backports(v.1.2.1)*, *httpuv(v.1.6.1)*, *rsconnect(v.0.8.18)*, *tensorA(v.0.36.2)*, *tools(v.4.0.3)*, *bookdown(v.0.22)*, *cubature(v.2.0.4.2)*, *ellipsis(v.0.3.2)*, *jquerylib(v.0.1.4)*, *proxy(v.0.4-26)*, *MCMCglmm(v.2.32)*, *ggridges(v.0.5.3)*, *base64enc(v.0.1-3)*, *ps(v.1.6.0)*, *prettyunits(v.1.1.1)*, *zoo(v.1.8-9)*, *haven(v.2.4.1)*, *fs(v.1.5.0)*, *magrittr(v.2.0.1)*, *data.table(v.1.14.0)*, *openxlsx(v.4.2.4)*, *colourpicker(v.1.1.0)*, *lmtest(v.0.9-38)*, *reprex(v.2.0.0)*, *mvtnorm(v.1.1-2)*, *matrixStats(v.0.59.0)*, *hms(v.1.1.0)*, *shinyjs(v.2.0.0)*, *mime(v.0.11)*, *evaluate(v.0.14)*, *xtable(v.1.8-4)*, *shinystan(v.2.5.0)*, *rio(v.0.5.27)*, *readxl(v.1.3.1)*, *gridExtra(v.2.3)*, *rstantools(v.2.1.1)*, *compiler(v.4.0.3)*, *maps(v.3.3.0)*, *V8(v.3.4.2)*, *crayon(v.1.4.1)*, *minqa(v.1.2.4)*, *htmltools(v.0.5.1.1)*, *corpcor(v.1.6.9)*, *mgcv(v.1.8-36)*, *later(v.1.2.0)*, *RcppParallel(v.5.1.4)*, *lubridate(v.1.7.10)*, *DBI(v.1.1.1)*, *formatR(v.1.11)*, *dbplyr(v.2.1.1)*, *boot(v.1.3-28)*, *car(v.3.0-11)*, *cli(v.3.0.0)*, *mitools(v.2.4)*, *parallel(v.4.0.3)*, *igraph(v.1.2.6)*, *pkgconfig(v.2.0.3)*, *foreign(v.0.8-81)*, *laeken(v.0.5.1)*, *sp(v.1.4-5)*, *xml2(v.1.3.2)*, *dygraphs(v.1.1.1.6)*, *svglite(v.2.0.0)*, *bslib(v.0.2.5.1)*, *webshot(v.0.5.2)*, *estimability(v.1.3)*, *rvest(v.1.0.0)*, *callr(v.3.7.0)*, *digest(v.0.6.27)*, *rmarkdown(v.2.9)*, *cellranger(v.1.1.0)*, *curl(v.4.3.2)*, *shiny(v.1.6.0)*, *gtools(v.3.9.2)*, *nloptr(v.1.2.2.2)*, *lifecycle(v.1.0.0)*, *nlme(v.3.1-152)*, *jsonlite(v.1.7.2)*, *carData(v.3.0-4)*, *fansi(v.0.5.0)*, *pillar(v.1.6.1)*, *lattice(v.0.20-44)*, *loo(v.2.4.1)*, *fastmap(v.1.1.0)*, *httr(v.1.4.2)*, *DEoptimR(v.1.0-9)*, *pkgbuild(v.1.2.0)*, *survival(v.3.2-11)*, *glue(v.1.4.2)*, *xts(v.0.12.1)*, *zip(v.2.2.0)*, *shinythemes(v.1.2.0)*, *pander(v.0.6.4)*, *class(v.7.3-19)*, *stringi(v.1.7.2)*, *sass(v.0.4.0)*, *e1071(v.1.7-7)* and *ape(v.5.5)*

# References

Dingemanse, N. J., & Dochtermann, N. A. (2013). Quantifying individual variation in behaviour: Mixed-effect modelling approaches. *Journal of Animal Ecology*, *82*, 39–54.

Enders, C. K., Mistler, S. A., & Keller, B. T. (2016). Multi-level multiple imputation: A review and evoluation of joint modeling and chained equations imputation. *Psychological Methods*, *21*, 222–240.

Grund, Lüdtke, S., & Robitzsch, A. (2016). Multiple imputation of missing covariate values in multilevel models with random slopes: A cautionary note. *Behavior Research Methods*, *48*, 640–649.

Grund, L¨udtke, S., & Robitzsch, A. (2018). Multiple imputation of missing data at level 2: A comparison of fully conditional and joint modeling in multilevel designs. *Journal of Educational and Behavioral Statistics*, *doi.org/10.3102/1076998617738087*.

Grund, S., Lüdtke, O., & Robitzsch, A. (2017). Multiple imputation of missing data for multilevel models. *Organizational Research Methods*, *21*, 111–149.

Khwaja, N., Preston, S. A. J., Hatchwell, B. J., Briskie, J. V., Winney, I. S., & Savage, J. L. (2017). Flexibility but no coordination of visits in provisioning riflemen. *Animal Behaviour*, *125*, 25–31.

Liebl, A. L., Browning, L. E., & Russell, A. F. (2016). Manipulating carer number versus brood size: Complementary but not equivalent ways of quantifying carer effects on offspring. *Behavioral Ecology*, *27*, 1247–1254.

Lüdtke, O., Robitzsch, A., & Grund, S. (2017). Multiple imputation of missing data in multilevel designs: A comparison of different strategies. *Psychological Methods*, *22*, 141–165.

Noble, D. W. A., Stenhouse, V., & Schwanz, L. E. (2018). Developmental temperatures and phenotypic plasticity in reptiles: A systematic review and meta-analysis. *Biological Reviews*, *93*, 72–79.

Resche-Rigon, M., & White, I. R. (2018). Multiple imputation by chained equations for systematically and sporadically missing multilevel data. *Stat Methods Med Res*, *27*, 1634–1649.

Réale, D., Garant, D., Humphries, M. M., Bergeron, P., Careau, V., & Montiglio, P.-O. (2010). Personality and the emergence of the pace-of-life syndrome concept at the population level. *Philisophical Transactions of the Royal Society B.*, *365*, 4051–4063.

Rhemtulla, M., Jia, F., Wu, W., & Little, T. D. (2014). Planned missing designs to optimize the efficiency of latent growth parameter estimates. *International Journal of Behavioral Development*, *38*, 423–434.

Sih, A., Bell, A. M., Johnson, J. C., & Ziemba, R. E. (2004). Behavioral syndromes: An integrative overview. *Quarterly Review of Biology*, *79*, 241–277.

Stamps, J., & Groothuis, T. G. G. (2010). The development of animal personality: Relevance, concepts and perspectives. *Biological Reviews*, *85*, 301–325.

van Buuren, S. (2018). *Flexible imputation of missing data*. Chapman & Hall CRC.

van Buuren, S., & Groothuis-Oudshoorn, K. (2011). mice: Multivariate imputation by chained equations in r. *Journal of Statistical Software*, *45*(3), 1–67. Retrieved from https://www.jstatsoft.org/v45/i03/
